# Comprehensive bioinformatics analysis of NOX4 as a biomarker for pan-cancer prognosis and immune infiltration

**DOI:** 10.18632/aging.205769

**Published:** 2024-04-25

**Authors:** Yuying Liu, Hua Huang, Xijun Yang, Danhe Huang, Xiongwei Wang, Mingyu Yuan, Lianqing Hong

**Affiliations:** 1 Department of Pathology, Nanjing Integrated Traditional Chinese and Western Medicine Hospital Affiliated with Nanjing University of Chinese Medicine, Nanjing, China; 2 Tianjin Medical University, Tianjin, China; 3 Department of Anesthesiology, Fudan University Shanghai Cancer Center, Shanghai, China

**Keywords:** pan-cancer analysis, bioinformatics, NADPH oxidase 4, tumor immune microenvironment, immune checkpoint

## Abstract

Background: NADPH oxidase 4 (NOX4) has been proven to be associated with the prognosis of tumors in multiple cancers and can serve as a potential immunotherapy target to provide new treatment options for various tumors. In this study, our aim is to conduct an in-depth investigation of NOX4 across a range of cancer types to determine the relationship between NOX4 and tumors.

Methods: Utilizing large-scale transcriptomic and clinical data from public databases, a systematic examination of NOX4 expression patterns was performed in pan-cancer cohorts. Survival analysis, methylation analysis, and correlation studies were employed to assess the diagnostic and prognostic significance of NOX4 in diverse cancer types. Additionally, an exploration of the relationship between NOX4 expression and immune infiltration across various tumors was conducted.

Results: The analyses unveiled a consistent upregulation of NOX4 expression in multiple cancer types relative to normal tissues, indicating its potential as a universal cancer biomarker. Elevated NOX4 expression significantly correlated with poor overall survival in several cancers. Furthermore, the study demonstrated a robust correlation between NOX4 expression and immune cell infiltration, signifying its involvement in the modulation of the tumor microenvironment.

Conclusions: This study imparts valuable insights into the potential applications of NOX4 in cancer research, highlighting its significance as a multifaceted biomarker with diagnostic, prognostic, and immunomodulatory implications across diverse malignancies.

## INTRODUCTION

Cancer is a complex group of diseases and remains a major cause of human mortality globally. It continues to pose a significant global health challenge, necessitating urgent research efforts and solutions [1, 2]. The current central focus of cancer research is on identifying effective biomarkers to facilitate early diagnosis of cancer, predict patient prognosis, and select appropriate treatment strategies [3].

NADPH oxidase 4 (NOX4) is one of the seven members of the Nox family (Nox1, Nox2, Nox3, Nox4, Nox5, Duox1, and Duox2), and Nox4 has been identified as one of the major sources of reactive oxygen species (ROS). Increased generation of ROS has been implicated in the pathogenesis of various diseases such as cancer and cardiovascular disease [4]. NOX4 is widely expressed in many different tissues and has a wider range of biological functions. As a major endogenous ROS source, NOX4 is involved in regulating multiple cellular functions, including cell proliferation, migration, and death [5, 6]. Dysregulation of NOX4 expression in cancer has been observed across a spectrum of malignancies [7, 8]. Initial studies indicate elevated NOX4 expression in tumors compared to adjacent normal tissues, suggesting a potential role in tumorigenesis [9]. The multifaceted engagement of NOX4 in cancer has generated interest in its potential as a diagnostic and prognostic biomarker, as well as its influence on the tumor microenvironment through interactions with the immune system. The diagnostic potential of NOX4 is rooted in its abnormal expression in cancer tissues, including but not limited to breast cancer, lung cancer, colorectal cancer, and pancreatic cancer [10–13]. Although the role of NOX4 has been identified in some tumors, the current study lacks a comprehensive study of NOX4 in all tumors.

The prognostic significance of NOX4 in cancer is also gradually gaining attention. Several studies have proposed a correlation between high NOX4 expression and adverse clinical outcomes, including reduced overall survival (OS) and disease-free survival in cancer patients [14]. However, the consistency of these findings across diverse cancer types and the underlying molecular mechanisms governing NOX4-mediated prognostic effects necessitate further exploration. Further exploration is needed to investigate the interaction between the expression of NOX4 and the immune microenvironment. More and more evidence suggest that NOX4 may influence the immune infiltration of tumors, as well as the recruitment and function of immune cells [15]. Studies have shown that NOX4 is essential for maintaining the immunosuppressive CAF phenotype in tumors. Pharmacological inhibition of NOX4 enhances immunotherapy by overcoming CAF-mediated CD8 T cell exclusion [7]. Understanding the relationship between NOX4 expression and immune infiltration is crucial for unraveling the immunomodulatory aspects of NOX4 in cancer progression. Nevertheless, the extent of NOX4’s involvement in different cancer types and its clinical implications have not been systematically explored.

In summary, this study aims to elucidate the multifaceted role of NOX4 in cancer by exploring its diagnostic potential, prognostic significance, and impact on immune infiltration in tumors. It seeks to provide insights for future experimental research and aid in the clinical translation of NOX4 as a tumor biomarker, thereby improving the diagnosis, prognosis, and selection of treatment options for cancers.

## MATERIALS AND METHODS

### Data acquisition and processing

The assessment of NOX4 expression involved a comprehensive examination across 34 distinct tumors and their corresponding normal tissues, utilizing datasets from The Cancer Genome Atlas (TCGA) and Genotype-Tissue Expression (GTEx) cohorts. The SangerBox web-based tool (http://sangerbox.com/) facilitated this analysis. In addition, the “Pathological Stage Plot” module of Gene Expression Profiling Interactive Analysis [16] was employed to scrutinize NOX4 expression levels in various pathological stages within selected TCGA tumors.

### NOX4-related gene enrichment analysis

For protein-protein interaction network associated with NOX4, the STRING website (https://string-db.org/) [17] was utilized. Additionally, the “Similar Gene Detection” module of GEPIA2 identified the top 100 NOX4-associated genes in TCGA tumors. Subsequently, the correlation analysis module of GEPIA2 explored the correlation between NOX4 and the top five NOX4-associated target genes. Pathway and process enrichment analysis for the identified top 100 NOX4-associated target genes was conducted using the Metascape web-based tool [18], with specific parameters set to P < 0.01, a minimum count of three for terms, and an enrichment factor > 1.5 for canonical pathways.

### Analysis of tumor immune and immunosuppressive cell infiltration

To assess the correlation between NOX4 expression and the infiltration of various immune cell types, the TIMER2 server [19] was employed. The impact of genetic and epigenetic alterations of NOX4 on dysfunctional T-cell phenotypes was evaluated using the QUERY module of the Tumor Immune Dysfunction and Exclusion (TIDE) algorithm [20].

### Epigenetic methylation analysis

The TCGA methylation module within the UALCAN interactive web resource was utilized to investigate differences in NOX4 methylation levels between tumor and paired normal tissues across various TCGA cancer types. Furthermore, the TIDE server was employed to explore the effect of NOX4 methylation on dysfunctional T-cell phenotypes and prognoses.

### Statistical analysis

Student’s t-test was applied to compare NOX4 expression levels between different groups, and the Wilcoxon rank-sum test was employed for non-normally distributed data. Pearson correlation analysis was used to evaluate the correlation between NOX4 expression and immune infiltration.

### Data and materials availability

Data from the TCGA and public databases were utilized and examined in the present investigation. For additional information on this study, please contact the corresponding author. The entire data needed to evaluate the findings can be found within this article.

## RESULTS

### Pan-cancer analysis of NOX4 expression

**Figure 1:**
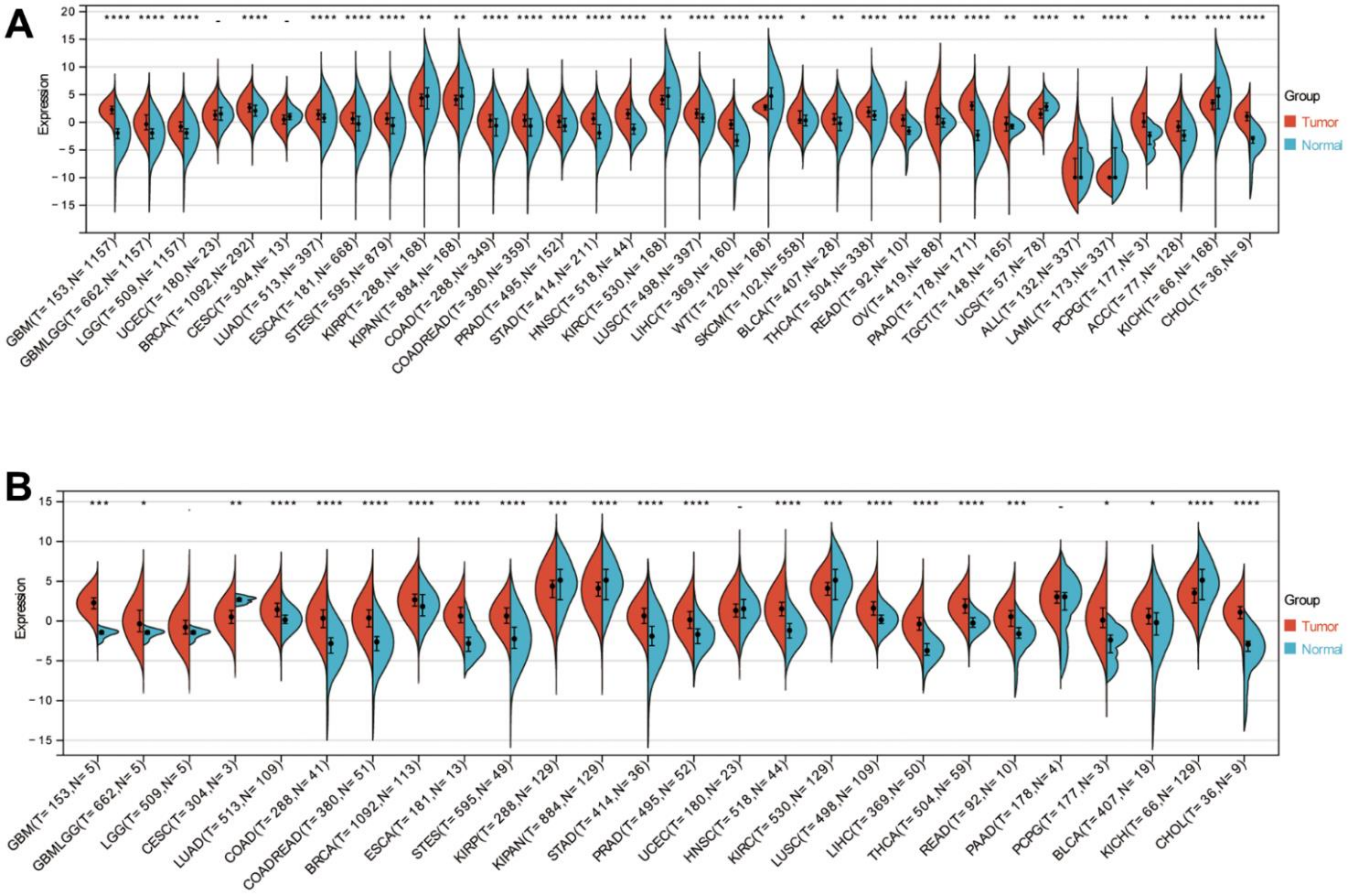
**NOX4 expression in cancers.** (**A**) The bar plot illustrates NOX4 mRNA expressions in various normal human tissues sourced from the TCGA and GTEx databases. (**B**) NOX4 mRNA expression levels in diverse cancer types based on TCGA databases; (*P < 0.05, **P < 0.01, ***P < 0.001, ****P < 0.0001).

A comprehensive pan-cancer analysis of NOX4 expression was conducted using data from TCGA and GTEx cohorts. The analysis revealed elevated NOX4 expression in the majority of tumors compared to their corresponding normal tissues (25 out of 34) (Figure 1A). A consistent pattern was observed when focusing solely on the TCGA database, with increased NOX4 expression evident in 19 out of 26 tumors (Figure 1B). These findings underscore the close association between NOX4 and the initiation and progression of diverse tumors.

### Prognostic implications of NOX4 expression

**Figure 2:**
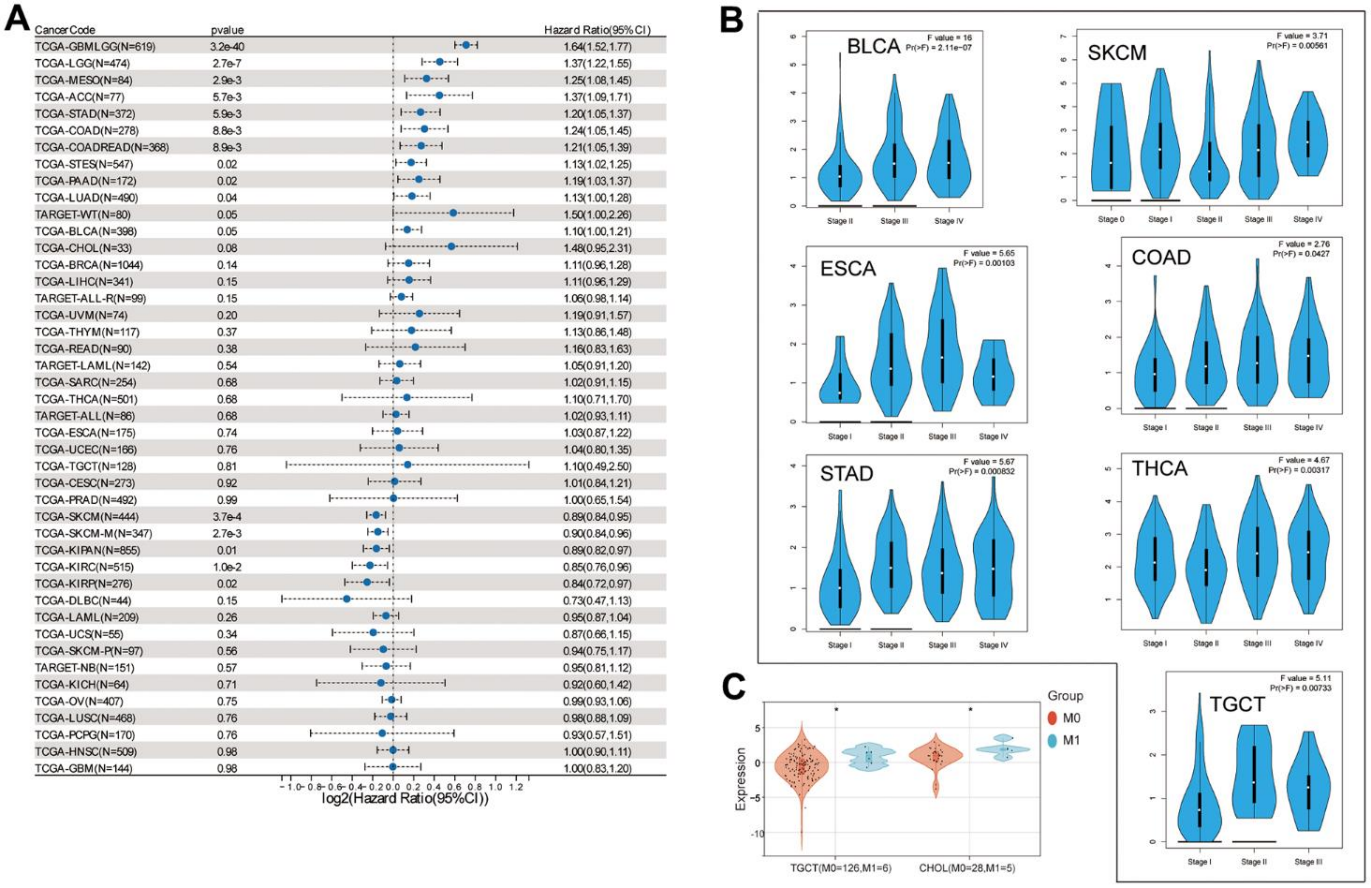
**Correlation of NOX4 expression with prognosis and clinical stages.** (**A**) Examination of the prognostic association of NOX4 expression in various cancer types utilizing SangerBox. (**B**) Investigation into the association of NOX4 gene expression levels with pathological stages. (**C**) Exploration of the relationship between NOX4 expression and metastasis; (*P < 0.05).

Further exploration of the correlation between NOX4 expression and patient outcomes indicated a consistent association between high NOX4 expression and poorer prognosis across several cancer types, including GBMLGG, LGG, MESO, ACC, STAD, COAD, COADREAD, STES, PAAD, LUAD, WT, and BLCA (Figure 2A). Notably, in SKCM, SKCM-M, KIPAN, KIRC, KIRP, patients with high NOX4 expression exhibited better OS. Additionally, NOX4 expression increased with higher tumor stages in BLCA, SKCM, ESCA, COAD, STAD, THCA, and TGCT (Figure 2B).

A detailed investigation highlighted significantly higher NOX4 expression in TGCT and CHOL among patients with metastases (Figure 2C).

### DNA methylation analysis

**Figure 3:**
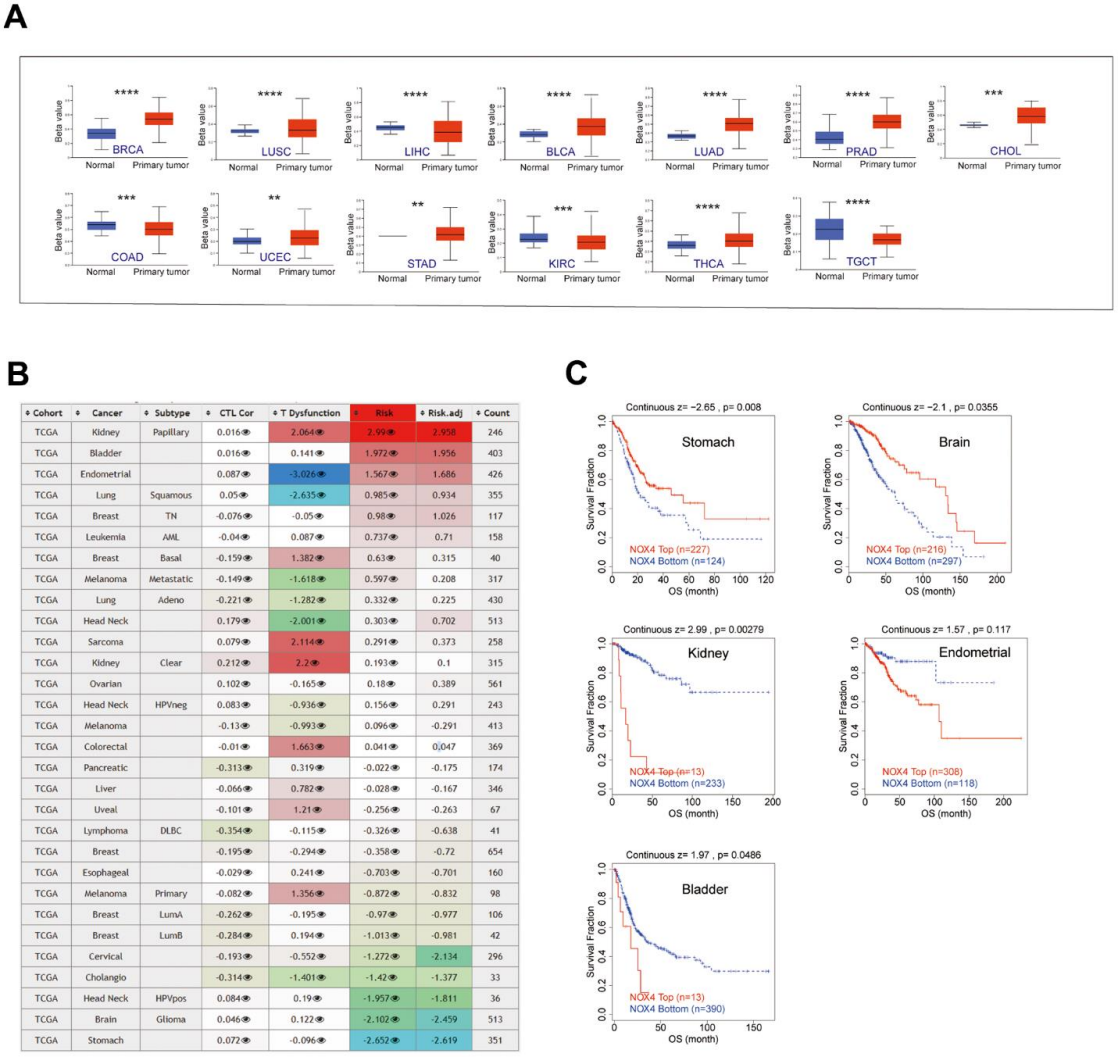
**Epigenetic methylation analysis.** (**A**) Boxplots depict the differential NOX4 methylation levels (beta values) across TCGA cohorts. (**B**) A heatmap displays the impact of NOX4 methylation on cytotoxic T-cell levels (CTLs), dysfunctional T-cell phenotypes, and risk factors within TCGA cohorts. (**C**) Kaplan-Meier curves compare overall survival differences between high and low NOX4 methylation levels, with statistically significant differences depicted; (**P < 0.01, ***P < 0.001, ****P < 0.0001).

Assessing DNA methylation alterations, crucial for the identification of diagnostic and prognostic biomarkers, revealed NOX4 hypermethylation in various cancer types, including BRCA, LUSC, BLCA, LUAD, PRAD, UCEC, STAD, THCA, and CHOL (Figure 3A). The consequences of NOX4 methylation status varied across cancers, with hypermethylation positively associated with patient risk and correlated with shorter OS in bladder, kidney and endometrial cohorts. Conversely, in brain and stomach cohorts, NOX4 hypermethylation was negatively associated with patient risk and correlated with longer OS (Figure 3B, 3C).

### Functional enrichment analysis

**Figure 4:**
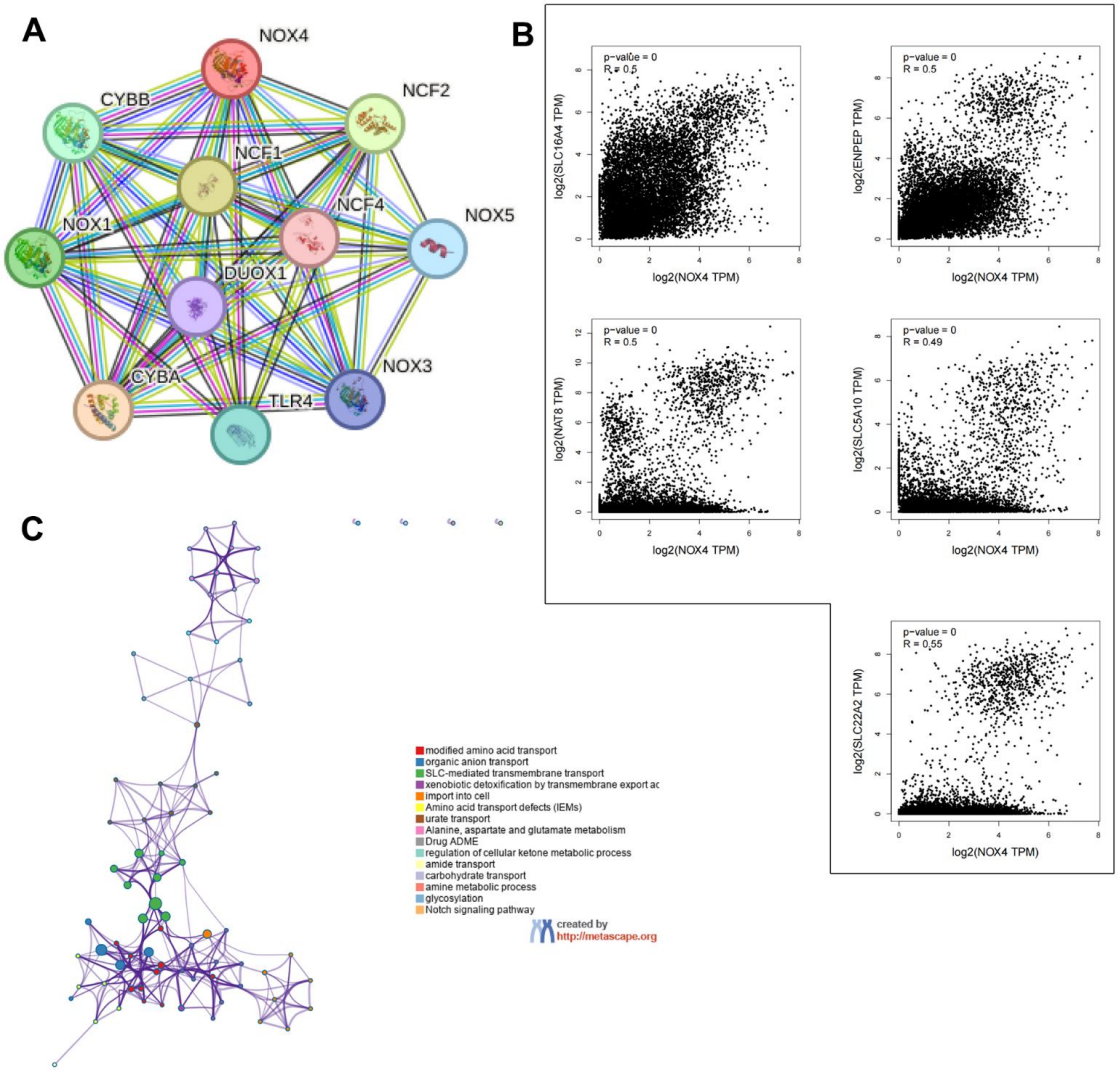
**Enrichment analysis of NOX4-related genes across pan-cancers.** (**A**) Identification of NOX4-binding proteins using the STRING tool. (**B**) Exploration of the top five NOX4-correlated genes across pan-cancers and their relationships with NOX4 expression analyzed through the GEPIA2 website. (**C**) Potential biological functions analysis of NOX4 by Metascape; presentation of enriched terms with a similarity >0.3 connected by edges.

Exploring NOX4-binding proteins through the STRING tool identified interactions with CYBB, NOX1, CYBA, DUOX1, TLR4, NOX3, NOX5, NCF1, NCF2, and NCF4 (Figure 4A). Correlation analysis of gene expression data from TCGA highlighted SLC16A4, ENPEP, NAT8, SLC5A10, and SLC22A2 as the top five genes significantly correlated with NOX4 expression (Figure 4B). The top 100 NOX4-associated genes were significantly associated with cancer-related signaling pathways, such as modified amino acid transport, organic anion transport, and SLC-mediated transmembrane transport (Figure 4C). These findings suggest a potential role for NOX4 in modulating cancer metabolism.

### NOX4 is related to immunity

**Figure 5:**
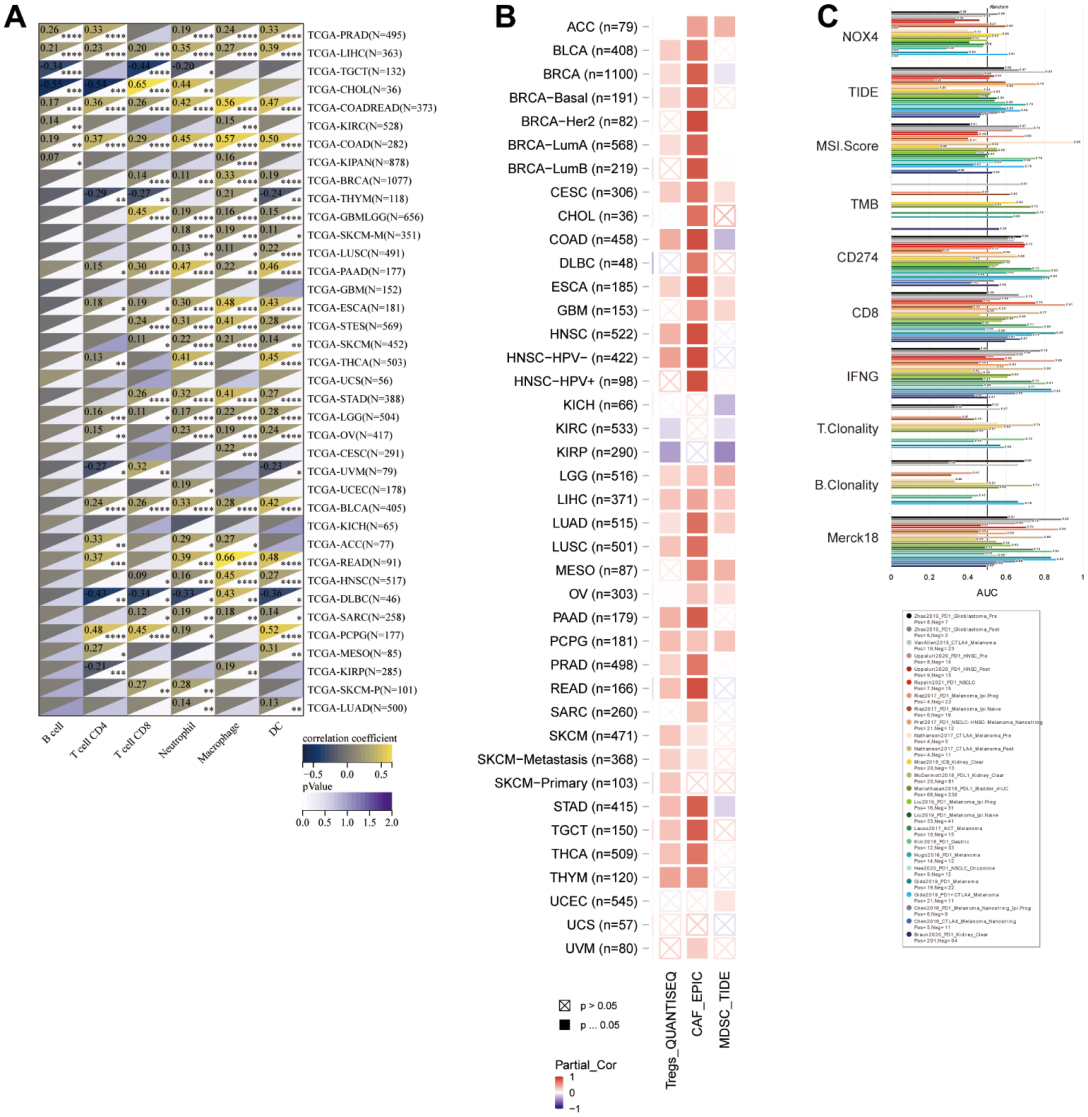
**Correlations between NOX4 expression and immune cell infiltration levels.** (**A**) A heatmap displays the correlations between NOX4 expression and infiltration levels of six immune cell types in TCGA cohorts. (**B**) Another heatmap depicts the correlations between NOX4 expression and infiltration levels of three immunosuppressive cell types in TCGA cohorts. (**C**) A bar plot compares the biomarker relevance of NOX4 with standardized cancer immune evasion biomarkers in immune checkpoint blockade (ICB) subcohorts, including cancer-associated fibroblasts (CAFs), myeloid-derived suppressor cells (MDSCs), and regulatory T cells (Tregs); (*P < 0.05, **P < 0.01, ***P < 0.001, ****P < 0.0001).

Investigating the relationship between NOX4 expression and immune cell infiltration in the tumor microenvironment revealed a significant positive correlation with six immune cell types in most tumor types, excluding DLBC, UVM, THYM, and TGCT (Figure 5A). Additionally, NOX4 expression was positively correlated with the infiltration abundance of cancer-associated fibroblasts (CAFs) in most tumors (Figure 5B). Comparing NOX4 with established biomarkers for predicting response outcomes and OS in immune checkpoint blockade (ICB) sub-cohorts demonstrated an area under the receiver operating characteristic curve (AUC) of >0.5 in 7 of 18 ICB sub-cohorts (Figure 5C).

### Correlation of NOX4 expression with immune checkpoint genes

**Figure 6:**
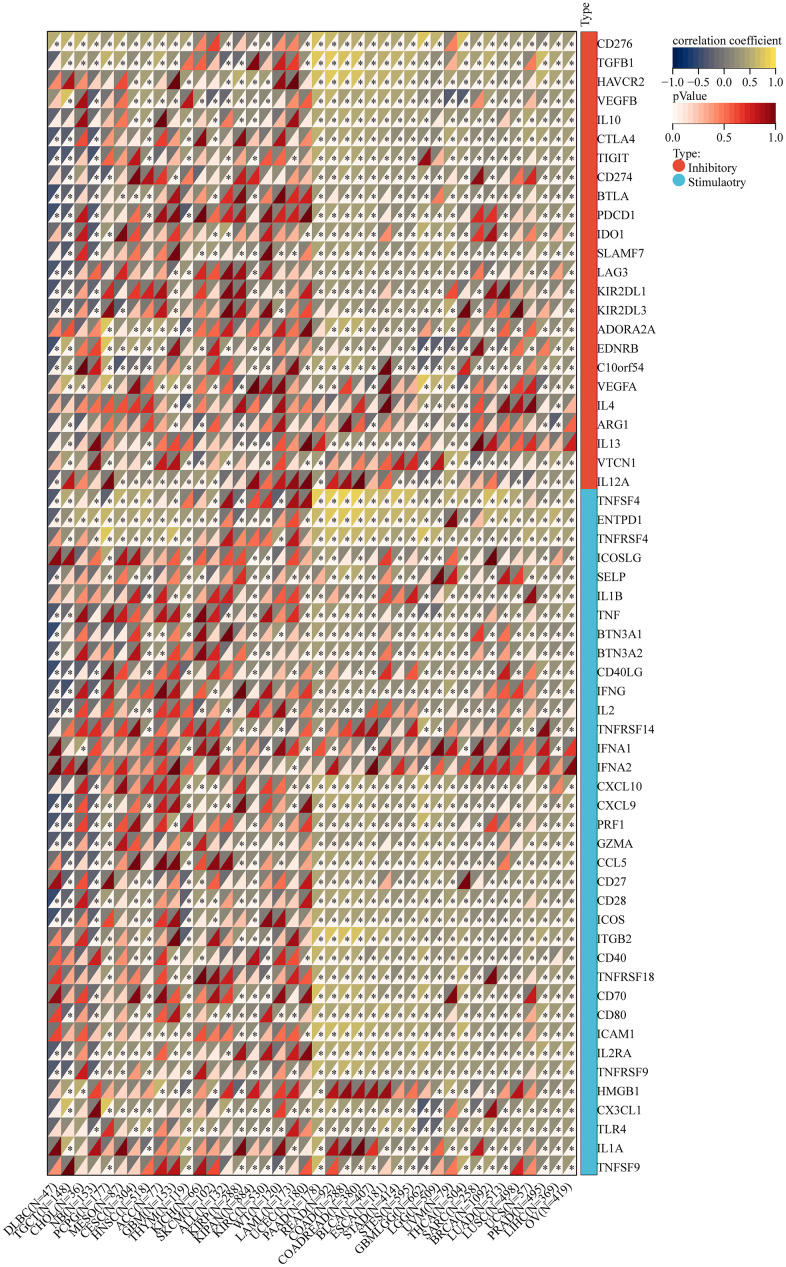
Relationship between NOX4 mRNA expression and immune checkpoints in multiple cancers; (*P < 0.05).

Further investigation into the correlation between NOX4 expression and immune checkpoint genes across 40 cancer types revealed a close association with common immune checkpoints, such as PD-L1 and CTLA-4 (Figure 6). This implies a potential role for NOX4 in regulating immune checkpoint gene expression, suggesting its involvement in immune evasion mechanisms of cancer cells.

## DISCUSSION

Cancer’s complex landscape demands continual exploration of novel therapeutic targets to improve current treatment outcomes. As a member of the NADPH oxidase family, NOX4 plays a crucial role in the generation and regulation of ROS, making it a potential new target for cancer therapy [21]. Previous studies have shown that aberrant expression of NOX4 in tumor cells leads to elevated cellular ROS levels, promoting tumorigenesis. This abnormal expression is closely associated with accelerated cell proliferation, angiogenesis, and resistance to apoptosis [22]. Therefore, a thorough exploration of the functional significance and specific mechanisms of NOX4 in cancer is crucial for the development of new treatment modalities. By elucidating the role of NOX4 in tumorigenesis, researchers aim to reveal new avenues for precision medicine, offering innovative approaches to address the multifaceted challenges posed by cancer. Studies suggest that targeting NOX4 as a potential druggable target to disrupt redox balance within cancer cells can inhibit cancer growth by mitigating the pro-tumorigenic effects associated with increased ROS production [23]. Continuous overexpression of NOX4 has been observed in breast cancer, colorectal cancer, clear cell renal cell carcinoma, and lung cancer compared to normal tissues, suggesting its potential as a universal cancer biomarker. Moreover, elevated levels of NOX4 expression are associated with poorer prognosis, manifested as shorter OS. Additionally, its correlation with advanced tumor stages across various cancers suggests a role for NOX4 in driving tumor progression.

Exploration of NOX4’s epigenetic regulation through DNA methylation analysis provides valuable insights. Hypomethylation of NOX4 has been observed in various cancer types, including breast cancer, lung squamous cell carcinoma, bladder cancer, lung adenocarcinoma, prostate cancer, endometrial cancer, gastric cancer, thyroid cancer, and gallbladder cancer, suggesting that epigenetic modifications may contribute to its dysregulation in cancer. Intriguingly, hypermethylation of NOX4 is associated with a lower risk and longer OS in specific cancer cohorts, highlighting the complex interplay between epigenetic regulation and clinical outcomes.

Functional enrichment analysis reveals potential roles for NOX4 in modulating cancer metabolism through its interaction with proteins involved in cancer-related signaling pathways such as CYBB, NOX1, CYB1, DUOX1, TLR4, NOX3, NOX5, NCF1, NCF2, and NCF4. This suggests that NOX4 may have multifunctional roles beyond its canonical function in redox homeostasis. The positive correlation between NOX4 expression and immune cell infiltration levels in the tumor microenvironment adds complexity to its role in cancer progression. Furthermore, the association with established biomarkers for predicting immune checkpoint blockade response outcomes positions NOX4 within the intricate network of immune responses in cancer.

In conclusion, this comprehensive analysis deepens our understanding of NOX4 in cancer, highlighting its diverse roles in gene expression, diagnostic potential, prognostic implications, epigenetic regulation, functional associations, and immune. The multifaceted involvement of NOX4 in cancer underscores its significance as a potential target for further research and clinical exploration.
